# Detection of *Leishmania* DNA in wild foxes and associated ticks in Patagonia, Argentina, 2000 km south of its known distribution area

**DOI:** 10.1186/s13071-016-1515-4

**Published:** 2016-04-28

**Authors:** Javier Millán, Alejandro Travaini, Stefania Zanet, José Vicente López-Bao, Anna Trisciuoglio, Ezio Ferroglio, Alejandro Rodríguez

**Affiliations:** Facultad de Ecología y Recursos Naturales, Universidad Andres Bello, Santiago, Chile; Centro de Investigaciones Puerto Deseado, UNPA-CONICET, CC 238, 9050 Puerto Deseado, Santa Cruz Argentina; Department of Animal Production, Epidemiology and Ecology, University of Turin, Grugliasco, TO Italy; Research Unit of Biodiversity (UO/CSIC/PA), Oviedo University, Mieres, Spain; Grimsö Wildlife Research Station, Department of Ecology, Swedish University of Agricultural Sciences (SLU), SE-730 91 Riddarhyttan, Sweden; Department of Conservation Biology, Estación Biológica de Doñana - CSIC, Américo Vespucio s/n, 41092 Sevilla, Spain

**Keywords:** Kinetoplastida, *Leishmania infantum*, *Lycalopex griseus*, *Lycalopex culpaeus*, Sylvatic cycle, Wildlife

## Abstract

**Background:**

Zoonotic Visceral Leishmaniasis (ZVL) is a vector-borne disease affecting humans and other mammals and caused by the protozoan parasite *Leishmania* (*Leishmania*) *infantum* (syn. *L. chagasi*), belonging to the *L. donovani* complex. The regions in Northern Argentina (above 32 °S) are its southern distribution limit in South America.

**Results:**

We detected *Leishmania* sp. DNA (most likely belonging to the *L. donovani* complex) in 37.5 % of 32 grey foxes (*Pseudalopex griseus*) captured in Argentinean Patagonia (48°S and 50°S). Eleven monosexual pools of *Amblyomma tigrinum* ticks from eight different foxes (six grey foxes and two culpeo foxes *P. culpaeus*) were also positive. The southernmost known distribution limit for *L. infantum,* and the southernmost reported capture of a phlebotominae, had previously been 2000 and 750 km north of our study area, respectively.

**Conclusions:**

This finding is significant because it markedly extends the distribution area of leishmaniasis; supports the existence of a sylvatic cycle in the absence of dogs; and has implications in transmission, indicating that either sand fly distribution is broader than currently thought or non-sand fly *Leishmania* maintenance is possible. Additional molecular, parasitological, epidemiological and entomological studies are still needed.

## Background

Zoonotic Visceral Leishmaniasis (ZVL) is a vector-borne disease affecting humans and other mammals and caused by the protozoan parasite *Leishmania* (*Leishmania*) *infantum* (syn. *L. chagasi*), belonging to the *L. donovani* complex. It is believed that the parasite was brought to South America by European immigrants, perhaps many times, spreading rapidly due to migration, urbanization and trade [1, 2]. The current known distribution area in South America comprises most of Brazil, Venezuela, Paraguay, Northern Argentina, western Bolivia, eastern Peru and other minor foci north of these areas. The regions in Northern Argentina (above 32°S) are its southern distribution limit [3].

ZVL is a serious public health problem and its primary reservoir is the dog [4]. ZVL also causes dog morbidity and mortality in areas where it is endemic [5]. Disease incidence in endemic areas has increased, suggesting that existing control measures consisting of vector control and dog culling have not been effective. To explain this lack of effectiveness, the existence of alternative reservoir hosts has been proposed among other factors [4]. The possibility that peri-domestic and sylvatic transmission cycles operate concurrently, involving different primary reservoir species (e.g. a domestic and a wild host, respectively) with a link between the two cycles, has also been suggested [4]. Wild carnivores are among those species suspected of serving as sylvatic reservoirs. In endemic areas of South America, the crab-eating fox (*Cerdocyon thous*) has long been known to have a high prevalence of infection in some areas of Brazil [6], including in a non-endemic area [7], suggesting an independent enzootic fox cycle. In another endemic region, the Mediterranean Basin, a high prevalence of *L. infantum* has been confirmed in several species of wild carnivores [8].

Most of the studies enumerated above were carried out in human-dominated landscapes where dogs are abundant and act as primary reservoirs of *Leishmania*. Therefore, it is difficult to clearly demonstrate the capacity of a wild species to maintain the parasite in a purely sylvatic cycle. The aim of the present study was to determine the presence of *Leishmania* sp. in an abundant wild carnivore inhabiting a remote, non-endemic area of South America were dogs are scarce and sand flies are not known to be present.

## Methods

### Field methods

**Fig. 1:**
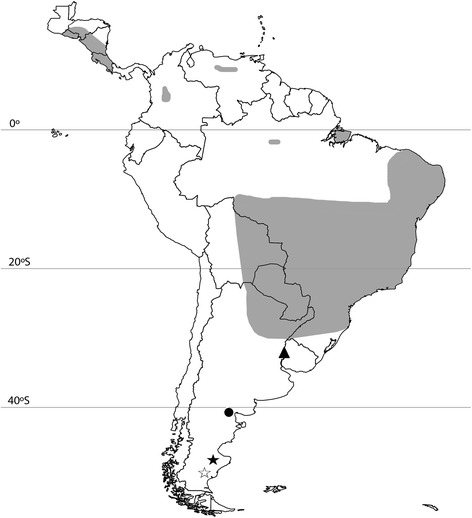
Map of South America, showing the accepted distribution area of *Leishmania infantum* (*grey area*) [3], the southernmost record of a Phlebotominae (*black circle*) [19], the southernmost record of a competent vector (*black triangle*) [31], the Bosques Petrificados National Monument (*black star*), and the Monte León National Park (*white star*)

Foxes were captured from 2010 to 2013 in two study areas: Monumento Natural Bosques Petrificados National Park (MNBP; 47°58'S, 67°97'W), and Monte León National Park (ML; 50°14′S, 69°00′O), both in Santa Cruz province, Argentinean Patagonia (Fig. 1, Table 1). The dominant habitat is shrub-steppe with < 50 % cover. The climate is dry and cold, with frequent frosts. The mean annual temperature is 10 °C (ranging from -10 °C to 30 °C), and annual rainfall ranges from 100 to 300 mm. Dogs are very rare in the study areas, with few individuals in some ranches surrounding the parks. Unpublished preliminary data estimated South American grey fox (*Pseudalopex griseus*; syn. *Lycalopex griseus*) density at around 0.3 foxes/100 ha, and culpeo fox (*P. culpaeus*) density at around 0.1 foxes/100 ha (A. Travaini and A. Rodríguez, unpublished data). The grey fox is widespread in plains and mountains on both sides of the Andes in Chile and Argentina [9], whereas the culpeo fox is distributed throughout the Andes and hilly regions of South America from Colombia to Tierra del Fuego [10]. We captured 34 free-living foxes, including 32 grey and 2 culpeo foxes. Foxes were caught between November 2010 and October 2013 with Oneida Victor #1.5 soft-catch coil spring traps (Cleveland, OH, USA), anaesthetized with a combination of tiletamine and zolazepam (Zoletil, Virbac, Spain). Blood obtained from the cephalic vein was either applied (100 μl) to FTA™ Nucleic Acid Collection Cards (Whatman, Maidstone, Kent, UK), air-dried and stored in sealed plastic bags or kept in 95 % ethanol (Table 1) until further processing. Ticks were retrieved from nine of the captured foxes and stored in ethanol until analysis. Foxes were released at the capture site. For one culpeo fox, only ticks and no blood was obtained. All analyzed ticks were adults of the species *Amblyomma tigrinum* (Table 1). Identification was carried out using standard morphological criteria [11].

**Fig. 2:**
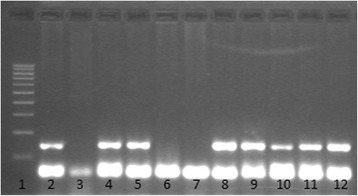
*Leishmania infantum* specific PCR amplifying a 145 bp fragment of kDNA. Lane 1: 100 bp low ladder (Sigma-Aldrich); Lane 2: PCR positive control; Lane 3: PCR negative control; Lanes 4–12: Grey foxes (7 positive and 2 negative)

**Table 1:** Foxes (*Pseudalopex* spp.) and ticks analyzed for the presence of *Leishmania* DNA in  Patagonia, Argentina

Ref.	Species	Location^a^	Date	Blood preservation	PCR result^b^		Ticks (*Amblyomma tigrinum*)^c^			
					Protocol 1	Protocol 2	Females	PCR Result	Males	PCR Result
ZG1	*P. griseus*	MNBP	26/11/2010	FTA card	**Positive**	**Positive**	5	**Positive**	8	Negative
ZG2	*P. griseus*	MNBP	26/11/2010	FTA card	**Positive**	**Positive**	1	**Positive**	2	Negative
ZG3	*P. griseus*	MNBP	29/11/2010	FTA card	**Positive**	Negative	10	Negative	17	Negative
ZG4	*P. griseus*	MNBP	29/11/2010	FTA card	**Positive**	Negative	5	**Positive**	6	**Positive**
ZG5	*P. griseus*	MNBP	02/12/2010	FTA card	**Positive**	Negative	2	**Positive**	2	**Positive**
ZC1	*P. culpaeus*	MNBP	20/02/2011	FTA card	Negative	Negative	7	Negative	6	**Positive**
ZG6	*P. griseus*	MNBP	11/04/2011	FTA card	**Positive**	**Positive**	0	-	1	**Positive**
ZG7	*P. griseus*	MNBP	11/04/2011	FTA card	**Positive**	Negative	1	Negative	1	**Positive**
ZG8	*P. griseus*	MNBP	12/04/2011	FTA card	Negative	Negative	0	-	0	-
ZC2	*P. culpaeus*	MNBP	14/04/2011	-	-	-	1	**Positive**	0	-
ZG10	*P. griseus*	MNBP	16/04/2011	FTA card	**Positive**	Negative	0	-	0	-
ZG11	*P. griseus*	MNBP	18/04/2011	FTA card	Negative	Negative	0	-	0	-
ZG12	*P. griseus*	MNBP	02/04/2012	FTA card	Negative	-	0	-	0	-
ZG13	*P. griseus*	MNBP	02/04/2012	FTA card	Negative	-	1	**Positive**	0	-
ZG14	*P. griseus*	MNBP	05/04/2012	FTA card	Negative	-	0	-	0	-
ZG15	*P. griseus*	MNBP	06/04/2012	FTA card	Negative	-	0	-	0	-
ZG16	*P. griseus*	MNBP	08/04/2012	FTA card	**Positive**	-	0	-	0	-
ZG17	*P. griseus*	MNBP	16/04/2012	FTA card	Negative	-	0	-	0	-
ZG18	*P. griseus*	MNBP	17/04/2012	FTA card	Negative	Negative	0	-	0	-
ZG19	*P. griseus*	MNBP	17/04/2012	FTA card	Negative	Negative	0	-	0	-
ZG20	*P. griseus*	MNBP	19/04/2012	FTA card	Negative	Negative	0	-	0	-
ZG21	*P. griseus*	MNBP	23/08/2012	FTA card	-	Negative	0	-	0	-
ZG22	*P. griseus*	MNBP	14/09/2012	FTA card	-	Negative	0	-	0	-
ZG23	*P. griseus*	MNBP	15/09/2012	FTA card	-	Negative	0	-	0	-
ZG24	*P. griseus*	MNBP	17/09/2012	FTA card	-	Negative	0	-	0	-
ZG25	*P. griseus*	MNBP	18/09/2012	FTA card	-	Negative	0	-	0	-
ZG26	*P. griseus*	ML	28/09/2013	95 % ethanol	-	**Positive**	0	-	0	-
ZG27	*P. griseus*	ML	28/09/2013	95 % ethanol	-	**Positive**	0	-	0	-
ZG28	*P. griseus*	ML	01/10/2013	95 % ethanol	-	Negative	0	-	0	-
ZG29	*P. griseus*	ML	01/10/2013	95 % ethanol	-	**Positive**	0	-	0	-
ZG30	*P. griseus*	ML	03/10/2013	95 % ethanol	-	Negative	0	-	0	-
ZG31	*P. griseus*	ML	03/10/2013	95 % ethanol	-	Negative	0	-	0	-
ZG32	*P. griseus*	ML	03/10/2013	95 % ethanol	-	Negative	0	-	0	-
ZG33	*P. griseus*	ML	03/10/2013	95 % ethanol	-	Negative	0	-	0	-

^a^MNBP: Monumento Natural Bosques Petrificados National Park; ML: Monte León National Park

^b^Protocol 1: perfomed at Facoltá di Medicina Veterinaria, Universitá degli Studi di Torino, Italia; Protocol 2: perfomed at VetGenomics, Barcelona, Spain

^c^Ticks were analyzed in pools. Results refer to the diagnostic by Protocol 1. Presence of *Leishmania* DNA in tick from ZG2 was also confirmed by Protocol 2

Positive cases are bold

### Laboratory methods

Samples were processed in two independent laboratories: PCR protocol 1 was performed at the Facoltá di Medicina Veterinaria, Universitá degli Studi di Torino, Italy (Lab 1); and Protocol 2, at VetGenomics, a veterinary molecular diagnostic company in Barcelona, Spain (Lab 2). DNA was independently extracted in Labs 1 and 2. Total genomic DNA was extracted from a single 2 mm punch of the FTA™ Cards following manufacturer’s instructions (GenSolve DNA Recovery Kit, Whatman, Maidstone, Kent, UK). For blood samples preserved in ethanol, 25 mg of blood was washed with 1 ml of PBS to eliminate ethanol. DNA was isolated using a DNeasy® Blood & Tissue Kit (Qiagen, California, USA) in a QIAcube according to manufacturer’s instructions. DNA was also extracted in Lab 1 from 17 monosexual pools of ticks using GenElute Mammalian Genomic Miniprep Kit (Sigma-Aldrich, St. Louis, MO, USA).

Two PCR protocols were performed. Protocol 1 used the *Leishmania donovani* (*sensu lato*)-specific primers (RV1-RV2) amplifying a 145 bp segment of the highly reiterated minicircles of kinetoplast DNA [12]. Protocol 1 was used for diagnosis of both blood and tick-extracted DNA. Amplification with primers mRv1 and mRv2 and agar gel verification were carried out as described in Ferroglio et al. [13]. One positive (total genomic DNA extracted from a pure culture of *L. infantum* promastigotes) and two negative controls were included in each PCR assay. Positive PCR products were purified using NucleoSpin® Gel and PCR Clean-up kits (Macherey-Nagel GmbH & Co. KG, Düren, Germany) and sequenced to confirm PCR results (BMR Genomics, Padua, Italy).

Protocol 2 consisted of a real-time PCR using a set of primers targeting a 121 bp segment of a different region of the parasite kinetoplast minicircle, namely LEISH-1 and LEISH-2, following Francino et al. [14]. Positive PCR products were purified and sequenced using BigDye v3.1 Kit (Live Technologies, Karlsruhe, Germany) using the same primers as in the PCR.

## Results

Twelve grey foxes (37.5 %) were positive for kinetoplast DNA by at least one of the protocols. Nine were positive by protocol 1 (Fig. 2), and six by protocol 2, with three foxes positive by both protocols 1 and 2. Readable sequences were obtained from four of the positive cases resulting from protocol 1, and two from protocol 2. BLAST analysis confirmed the PCR results (EMBL Nucleotide Sequence Database accession numbers: HF563611–HF563614 and LN794244). The obtained sequences showed the highest identity with *L. infantum* (four cases) and *L. major* (two cases) (Table 2). The single culpeo fox was negative. External lesions compatible with leishmaniasis were not observed.

**Table 2:** Sequences showing the highest identity with the sequences obtained in the present study

Reference	Obtained by	Sequence accession number	Name of the sequence showing the max identity	Host, country	% identity	Sequence accession number
ZG2	Protocol 2		*Leishmania major* strain MHOM/IL/67/LV561 minicircle, complete sequence; kinetoplast	Human, Iran	88	KM555288.1
Tick from ZG2	Protocol 2		*Leishmania donovani* kinetoplast minicircle DNA, isolate MHOM/BD/93/TANGAIL	Human, Bangladesh	91	AJ010085.1
ZG4 and ZG5	Protocol 1	HF563611 and HF563612	*Leishmania infantum* kinetoplast DNA, non-protein coding region, partial sequence, isolate: IranJWinf	Dog, Iran	96	AB678348.1
ZG6	Protocol 1	HF563613	*Leishmania infantum* kinetoplast DNA, non-protein coding region, partial sequence, isolate: IranJWinf	Dog, Iran	93	AB678348.1
			*Leishmania infantum* isolate MCAN/ES/98/10445 clone LinGpja_9 kinetoplast minicircle, complete sequence	Dog, Spain	93	EU437407
			*Leishmania donovani* isolate MHOM/SD/62/1S-Cl2D maxicircle, partial sequence; kinetoplast	Human, Sudan	93	FJ416603.1
	Protocol 2	LN794244	*Leishmania infantum* minicircle DNA, partial sequence	Human, Greece	88	AF027578.1
ZG10	Protocol 1	HF563614	*Leishmania major* (X239) kinetoplast DNA	Not reported	95	Z32845.1
			*Leishmania major* (X2312) kinetoplast DNA	Not reported	95	Z32844.1

Additionally, 11 monosexual pools of *A. tigrinum* (64.7 %) from eight different foxes were positive for *Leishmania* DNA by means of the PCR protocol 1, including ticks from one PCR-negative grey fox and the one culpeo fox for which blood was not available (Table 1). The presence of *Leishmania* DNA in one tick pool was confirmed with protocol 2 (Table 1) and sequenced, showing the maximum identity with *L. donovani* (Table 2).

## Discussion

This represents the detection of an infected mammalian host 2000 km south of the currently accepted southern boundary for the distribution of the parasite [15, 16]. The only member of the *L. donovani* complex known to be present in South America is *L. infantum* (syn. *L. chagasi*). This is in agreement with most of our BLAST results, including the sequence obtained from the positive tick, though *L. major* showed the highest identity in two cases. This is likely due to the fact that *L. infantum* and *L. major* share a high proportion of kinetoplast DNA because they both belong to the subgenus  *Leishmania* (*Leishmania*). Moreover, *L. major* is not present in South America. Therefore, we hypothesize that the detected DNA most likely indicates infection with *L. infantum* or closely related *Leishmania*. In any case, the obtained sequence homologies are too low to incriminate a *Leishmania* species and the actual identity of the detected parasite should be further investigated.

Our finding is significant for three main reasons. First, it markedly extends the geographical distribution of the parasite. Thus, *L. infantum* may be present in other undetected foci in southern South America, as was recently revealed in North America, where dogs from several US states and Canada had leishmaniasis [17]. We recommend that medical and veterinary practitioners from non-endemic areas include leishmaniasis when performing differential diagnoses because leishmaniasis may pass unnoticed [18].

Secondly, a role for wildlife in the epidemiology of leishmaniasis is further supported. The range of wild mammals in which *L. infantum* is detected is increasing, mainly amongst carnivores, rodents, marsupials, lagomorphs and even bats, some of which have been proven to be competent hosts by means of xenodiagnosis [4]. Recently, the existence of a sylvatic cycle in Northern Argentina was proposed in which the dogs would play the role of accidental host [19]. Given the scarcity of dogs in the study areas, with only a few dogs living on surrounding ranches, *L. infantum* appears to maintain a sylvatic cycle in Patagonia.

Thirdly, our finding has implications for *Leishmania* transmission. It is commonly accepted that phlebotomine sand fly transmission plays a central role in maintaining *L. infantum* infection because the spatial and temporal overlap of ZVL cases and the proven vector species show that sustained transmission does not generally occur in the absence of sand fly vectors [4]. Our results indicate that either sand fly distribution is broader than currently accepted, or that non-sand fly *Leishmania* maintenance is possible. Regarding sand fly distribution, the southernmost reported capture of a phlebotominae (*Lutzomyia oswaldoi*, without known vector capacity) was reported at 41 °S, 750 km north of MNBP [20]. Regarding *Leishmania* maintenance in the absence of sandflies, cases of autochthonous transmission of *L. infantum* have been described in northern Europe, where sand fly vectors are absent [21], and the sustained transmission of ZVL in foxhounds in non-endemic regions of North America was reported [22]. Autochthonous foci have also been found in parts of continental Europe and in the Alps [23, 24]. Recently, direct dog-to-dog contact was believed to be the most likely route of infection of *L. infantum* in the first autochthonous cases of canine leishmaniasis in New Caledonia because the affected dog was in close contact with two bitches imported from Spain [25]. Transmission by transfer of infected body fluids (e.g. by biting or sexual contact) [26] or congenitally [22], has been suggested to explain non-sand fly transmission. Congenital transmission to puppies has been confirmed experimentally [25]. Finally, *Leishmania* DNA has been found in ticks and fleas, suggesting their potential role in transmission ([27, 28]; this study). The ingestion of infected ticks is also an effective transmission route for certain tick-borne pathogens, and hamsters were experimentally infected with macerates of ticks collected from *L. infantum*-infected dogs [29]. Of course, the finding of infected ticks does not prove their role as a *Leishmania* vector, but it supports the presence of the parasite in the study area. Adult *A. tigrinum* feed predominantly on wild and domestic canids and occasionally on humans and others hosts, whereas nymphs typically infect rodents of the family Caviidae [30]. This family is represented in the study areas by the abundant southern mountain cavy (*Microcavia australis*). Thus, this rodent might be the reservoir of leishmaniasis in Patagonia and the role of this rodent in its epidemiology deserves further investigation.

## Conclusions

In summary, we have demonstrated the presence of *Leishmania* DNA in wild foxes and ticks in Patagonia. Potential false positives due to contamination are ruled out because DNA was independently extracted and amplified in two different laboratories. The fact that protocol 1 yielded more positive cases than Protocol 2 can be due to a more successful DNA extraction in Lab 1 or to differences in the sensibility of the PCR protocol. Nevertheless, Lab 2 was able to detect *Leishmania* DNA in blood samples preserved in ethanol that were not analyzed in Lab 1 (Table 1).

Our finding represents potential public and canine health implications that should be addressed by further research, including the complete identification of the *Leishmania* species, the infectiveness of foxes to sand flies, the presence of infection in domestic dogs and other potential reservoirs (i.e. cavies) in Patagonia, the occurrence of sand flies in the area, the potential role of other arthropods (fleas and ticks) as vectors, and other types of non-sand fly transmission, i.e. direct transmission between canids.

## Ethics statement

The present research complied with the regulations on animal experimentation and welfare issued by the European Union (Directive 86/609/CE). Specifically, capture and handling of foxes was approved by the competent authorities on bioethics and biosecurity under permit CEBA-EBD-2011-05b (Consejo Superior de Investigaciones Científicas, Spain), and the competent authorities on nature protection under permit 479-2011 (National Parks Administration, Argentina).
